# Point Prevalence Survey of Antimicrobial Use during the COVID-19 Pandemic among Different Hospitals in Pakistan: Findings and Implications

**DOI:** 10.3390/antibiotics12010070

**Published:** 2022-12-30

**Authors:** Zikria Saleem, Abdul Haseeb, Brian Godman, Narjis Batool, Ummara Altaf, Umar Ahsan, Faiz Ullah Khan, Zia Ul Mustafa, Muhammad Umer Nadeem, Muhammad Junaid Farrukh, Muhammad Mugheera, Inaam Ur Rehman, Asma Fareed Khan, Hamid Saeed, Mohammad Akbar Hossain, Mohamed Raafat, Rozan Mohammad Radwan, Muhammad Shahid Iqbal

**Affiliations:** 1 Department of Pharmacy Practice, Faculty of Pharmacy, Bahuddin Zakaria University, Multan 60800, Pakistan; 2 Department of Clinical Pharmacy, College of Pharmacy, Umm AL-Qura University, Makkah 21955, Saudi Arabia; 3 Centre of Medical and Bio-Allied Health Sciences Research, Ajman University, Ajman 346, United Arab Emirates; 4 School of Pharmacy, Sefako Makgatho Health Sciences University, Ga-Rankuwa, Pretoria 0208, South Africa; 5 Strathclyde Institute of Pharmacy and Biomedical Sciences, Strathclyde University, Glasgow G4 0RE, UK; 6 Australian Institute of Health Innovation, Center of Health Systems and Safety Research, Faculty of Medicine, Health and Human Sciences, Macquarie University, Sydney 2109, Australia; 7 Ghurki Trust Teaching Hospital, Lahore 54000, Pakistan; 8 Department of Infection Prevention and Control, Alnoor Specialist Hospital, Ministry of Health, Makkah 24382, Saudi Arabia; 9 Department of Pharmacy Administration and Clinical Pharmacy, Xi’an Jiaotong University, Xi’an 710061, China; 10 Discipline of Clinical Pharmacy, School of Pharmaceutical Sciences, Universiti Sains Malaysia, Gelugor 11800, Malaysia; 11 Department of Pharmacy Services, District Headquarter (DHQ) Hospital, Pakpattan 57400, Pakistan; 12 Punjab University College of Pharmacy, University of the Punjab, Lahore 54000, Pakistan; 13 Faculty of Pharmaceutical Sciences, UCSI University, Kuala Lumpur 56000, Malaysia; 14 Children’s Hospital and University of Child Health Sciences, Lahore 54000, Pakistan; 15 Department of Pharmacology and Toxicology, Faculty of Medicine in Al-Qunfudah, Umm Al-Qura University, Makkah 21955, Saudi Arabia; 16 Department of Pharmacology and Toxicology, College of Pharmacy, Umm AL-Qura University, Makkah 21955, Saudi Arabia; 17 Pharmaceutical Care Department, Alnoor Specialist Hospital, Ministry of Health, Makkah 24382, Saudi Arabia; 18 Department of Clinical Pharmacy, College of Pharmacy, Prince Sattam bin Abdulaziz University, Alkharj 11942, Saudi Arabia

**Keywords:** antimicrobial resistance, antimicrobial stewardship programs, antimicrobial utilization, COVID-19, irrational use of antibiotics, Pakistan, point prevalence survey

## Abstract

The COVID-19 pandemic has significantly influenced antimicrobial use in hospitals, raising concerns regarding increased antimicrobial resistance (AMR) through their overuse. The objective of this study was to assess patterns of antimicrobial prescribing during the current COVID-19 pandemic among hospitals in Pakistan, including the prevalence of COVID-19. A point prevalence survey (PPS) was performed among 11 different hospitals from November 2020 to January 2021. The study included all hospitalized patients receiving an antibiotic on the day of the PPS. The Global-PPS web-based application was used for data entry and analysis. Out of 1024 hospitalized patients, 662 (64.64%) received antimicrobials. The top three most common indications for antimicrobial use were pneumonia (13.3%), central nervous system infections (10.4%) and gastrointestinal indications (10.4%). Ceftriaxone (26.6%), metronidazole (9.7%) and vancomycin (7.9%) were the top three most commonly prescribed antimicrobials among surveyed patients, with the majority of antibiotics administered empirically (97.9%). Most antimicrobials for surgical prophylaxis were given for more than one day, which is a concern. Overall, a high percentage of antimicrobial use, including broad-spectrums, was seen among the different hospitals in Pakistan during the current COVID-19 pandemic. Multifaceted interventions are needed to enhance rational antimicrobial prescribing including limiting their prescribing post-operatively for surgical prophylaxis.

## 1. Introduction

In the early 1990s, infectious diseases were the most common cause of death globally, which was addressed by the improved availability of effective antimicrobials [1,2,3]. Overall, antimicrobials have played a significant role in the treatment of infectious diseases since their development [4]. 

However, the irrational use of antibiotics increases antimicrobial resistance (AMR), increasing morbidity, mortality and associated costs as well as decreasing their effectiveness [5,6,7,8,9,10]. AMR is now seen as a major challenge globally that urgently needs addressing [11,12,13]. Consequently, it is very important that antimicrobials are prescribed and dispensed rationally across all sectors of care [14,15]. Evidence-based optimized antimicrobial therapy is necessary in order to ensure their effectiveness, safety and cost effectiveness as well as reducing the potential for AMR [8,16]. 

The first case of COVID-19 caused by severe acute respiratory syndrome coronavirus 2, (SARS-CoV-2) was identified in Wuhan, China [17]. The virus subsequently spread exponentially, affecting more than 626 million people world-wide with over 6.5 million deaths by the end of October 2022 [18]. The first case of COVID-19 was confirmed in Pakistan on February 26, 2020 [19], with cases appreciably increasing after that [20,21,22]. Various preventive measures were instigated across countries to try and limit the spread of the virus prior to the availability of effective treatments including vaccines. Public health measures included social distancing, closure of borders, mask wearing, avoiding crowded areas, hand hygiene, and quarantining of close contacts [23,24,25].

Recent publications have suggested substantial overuse of antimicrobials during the COVID-19 pandemic despite limited bacterial or fungal co-infections [26,27,28,29,30]. In addition, concerns that COVID-19 surges will increase the prevalence of hospital acquired infections, with their impact on increased mortality and costs [31,32,33]. In their study, Guan et al. (2020) ascertained that 58% of 1099 patients with COVID-19 received intravenous antibiotics [34]. A study in Brazil documented that intravenous antibiotics were administered in 84.7% hospitalized patients suffering from COVID-19 [35]. Recent reviews have suggested that between 61.8% and 74.6% of patients hospitalized with COVID-19 were prescribed antibiotics despite limited bacterial or fungal co-infections [27,28,36,37]; with prescribing rates varying across countries [37]. However, lower rates of antimicrobial prescribing have been seen in some countries [38]. These high rates of antimicrobial utilization may be due to prevalent symptoms (cough, fever), anxiety regarding the COVID-19 outbreak and concerns with secondary infections especially as this increases mortality [30,39]. However, high inappropriate use of antimicrobials in patients with COVID-19 enhances AMR; consequently, such prescribing should be avoided where possible [39,40,41,42,43,44,45,46].

Data on general hospital antimicrobial use, prescribing and resistance patterns during the COVID-19 pandemic is typically limited in low- and middle-income countries (LMICs) including Pakistan, although this is changing [22,45,47,48,49,50]. Antimicrobial stewardship programs (ASPs) are increasingly being introduced across countries to improve future prescribing [51,52,53]. This increasingly includes LMICs despite the many challenges they face, which include a lack of knowledge regarding ASPs as well as available resources [54,55,56,57,58]. This trend will continue.

Point prevalence surveys (PPS) provide a reliable method to ascertain the quantity and quality of antimicrobial prescribing in hospitals. Subsequently, the findings help to determine appropriate ASPs to instigate to improve future antimicrobial use [59,60,61,62]. Consequently, this survey was undertaken among different hospitals across Pakistan to evaluate the prevalence of patients receiving antimicrobial agents whilst in hospital during the current pandemic. This included the patterns of antimicrobial prescribing in all hospitalized patients and not just those with COVID-19. 

This is important since according to WHO guidelines, no antimicrobial or antifungal drug should be prescribed in patients with mild or moderate COVID-19 until confirmation of pre-existing symptoms of bacterial or fungal co-infections [63]. The Dutch Working Party also suggested sputum and blood cultures should be taken in patients with COVID-19 before starting antimicrobial therapy and recommended subsequent de-escalation if pertinent based on culture results [64]. However, we are aware that there can be challenges with undertaking routine culture and sensitivity testing among hospitals in LMICs due to issues of available facilities and costs including patient co-payments [65]. 

Our findings will build on recent PPS studies conducted in Pakistan including patients with COVID-19 [22,66,67,68,69,70,71]. The findings can subsequently be used to suggest potential initiatives, including ASPs, which can be instigated in Pakistan to improve future antimicrobial prescribing to reduce current high rates of AMR. This is critical given the concerns that currently exist surrounding the national action plan to reduce AMR in Pakistan [72]. In addition, currently extensive inappropriate antimicrobial use across all healthcare sectors in Pakistan, which is exacerbated by limited ASP activities to date [71,72,73,74,75]. 

## 2. Results

In total, 11 hospitals with 1810 beds were included in this PPS. In these hospitals, 1024 (56.57%) patients were in-patients on the day of the survey. Out of these, 662 (64.64%) patients were prescribed one or more antimicrobial. The overall number of antimicrobials prescribed were 1191. This works out at 1.76 antimicrobials per patent. The majority of patients prescribed antimicrobials were male (57.2%). Of all the antimicrobials prescribed, 500 (42%) were prescribed in the medical departments of hospitals, with the vast majority administered parenterally (92.2% of all administrations). 

For the management of infections, an appreciable number of antimicrobials were given for community acquired infections (71.9%). 21.2% of antimicrobials were prescribed for surgical prophylaxis, with most patients (64.4%) receiving antimicrobials for more than one day. Of concern is that only one culture report was observed at the time of the survey, with almost all antibiotics prescribed empirically (97.9%). In an appreciable number of cases, the reasons for prescribing antibiotics were not documented in the patients’ medical files (89.8% of patients’ files) (Table 1). 

The total number of prescribed antimicrobials for systematic use was 1163 (97.6%), of whom 518 (43.5%) were cephalosporins and carbapenems (J01D) (Table 2). The other antimicrobials prescribed include antivirals (Table 2).

The most common clinical indications where antimicrobials were prescribed were pneumonia (13.3%), central nervous system infections (10.4%), gastrointestinal prophylaxis (10.4%), and obstetric and gynecological prophylaxis (10.2%). The top four most commonly prescribed antimicrobials were ceftriaxone –‘Watch’ antibiotic (26.6%), metronidazole—‘Access’ antibiotic (9.7%), vancomycin —‘Watch’ antibiotic (7.9%) and meropenem–Watch’ antibiotic (5.5%) (Table 3).

## 3. Discussion

We believe this is the first study undertaken in Pakistan that documents the prevalence of antimicrobial use among hospitals, including those with or without suspected or actual COVID-19, during the current pandemic. In our study, 64.64% of admitted patients had been prescribed at least one antimicrobial. However, it is difficult to compare this with other LMICs since most recent published studies, including systematic reviews, have concentrated on the prescribing of antimicrobials in patients with actual or suspected COVID-19 rather than all admitted patients. Typically, appreciable antimicrobial prescribing has been seen in patients admitted with COVID-19 across countries, averaging between 61.8% and 74.6% of all patients hospitalized with COVID-19 in recent studies despite limited bacterial or fungal co-infections [27,28,30,36,37,76]. However, lower rates of antimicrobial prescribing in patients with COVID-19 have been seen in some high-income countries. This includes Scotland at 38.3% [77], and Singapore at only 6.2% [38]. 

The average number of antimicrobials prescribed per patient in our study was 1.76. This was similar to the findings in Bahawal Victoria Hospital, Pakistan, at 1.4 per patient [78], and 1.64 per patent in a previous PPS study involving 13 hospitals in Pakistan [69]. However, both studies were pre-pandemic. Similar to a previous PPS study in Pakistan pre-pandemic, common indications for prescribing were prophylaxis for obstetrics and gynecology procedures as well as for gastrointestinal conditions [69]. There was similarly an appreciable administration of antimicrobials parenterally. This could be explained by the fact that among hospitalized patients admitted for COVID-19, injectable antimicrobials were prescribed to prevent deterioration despite limited bacterial or fungal co-infections [66]. This mirrors the situation seen among patients admitted to hospitals in Pakistan with COVID-19 [66,67].

The majority of antimicrobials in our study were prescribed for the treatment of infections. This may be due to a long stay among patients admitted to the hospital with COVID-19, increasing the risk of acquiring infections [79,80,81]. However, we could not investigate this further in our current study. Further studies are needed to assist with this as part of future quality improvement programs. Of concern is that the vast majority of antimicrobial prescriptions in our study were empiric appreciably increasing the chances of inappropriate prescribing. This though was similar to other studies in Pakistan as well as other LMICs in patients with or without COVID-19 [28,36,69,82,83,84,85,86]. In addition, an appreciable number of medical records (89.8%) were without any documentation of the rationale for prescribing. Increasing the documentation for the indication, as well as inserting start, stop and review dates, is an important step for good antimicrobial prescribing practices to limit inappropriate prescribing and reduce AMR [87,88,89,90]. There is also concern regarding current adherence to prescribing guidance in our study, with adherence to guidance increasingly seen as good-quality prescribing [60,91,92,93]. Adherence to guidelines should be increased by the ongoing development, dissemination, and subsequent monitoring of adherence to new national guidelines currently being developed via the National Institute of Health in Pakistan (Table S1).

To minimize the development of AMR, optimizing the course of treatment, including limiting the prescribing of antimicrobials post-operatively to reduce surgical site infections (SSIs), is important [16,94]. This includes promoting the prescribing of ‘Access’ antibiotics versus ‘Watch’ or ‘Reserve’ antibiotics from the ‘AWaRe’ list [95,96], with the over prescribing of ‘Watch’ antibiotics seen as problematic among LMICs [59]. In all hospitals, the cephalosporins, including ceftriaxone, were commonly prescribed antimicrobials because of their wide spectrum and safety. This is similar to previous studies conducted among LMICs [59,97,98,99]. A study in Bangladesh reported the same results on their COVID-19 wards [47]. In the present study, ceftriaxone was the most prescribed antimicrobial. This is a concern, with the prescribing of ceftriaxone generally increasing in Pakistan in recent years, similar to a study in the USA [100]. This is because such prescribing can increase extended spectrum beta lactamase (ESBL) producing multidrug resistant microbes [101]. The WHO have categorized ceftriaxone as a ‘Watch’ antibiotic in their AWaRe classification in view of the resistance potential [59,95,96], which should be reflected in any future national prescribing guidance produced in Pakistan. In addition, a key target for future ASPs in Pakistan should be to reduce inappropriate prescribing of ceftriaxone replaced by increased prescribing of pertinent ‘Access’ antibiotics. We have seen proactive ASPs and other approaches increasingly instigated among LMICs to improve future antimicrobial prescribing (Table S1) across sectors, which includes reviewing the prescribing of colistin—a ‘Reserve’ antibiotic [16,52,74,94,102,103,104,105], These examples (Table S1) provide future guidance to all key stakeholders in Pakistan.

We are aware of a number of limitations with this study. Firstly, we did not assess the appropriateness of antimicrobial use among all patients including those with COVID-19. This was hampered by the unavailability of local guidelines and lack of documentation in patients’ medical charts including a lack of CST findings. However, this is typical for PPS studies in LMICs. Similarly, we could only record information contained in the patients’ notes. We also did not differentiate between patients with or without COVID-19 in our study. This might have increased the prevalence rates of those without COVID-19. Finally, the number of hospitals actually taking part in the study were not fully representational of the current situation in Pakistan since a number of initially approached hospitals were not able to participate due to a variety of reasons. Nevertheless, we believe this study provides details of the patterns of antibiotic use among hospitals in Pakistan during COVID-19 and key areas to concentrate on for future ASPs. We will now be following this up. 

## 4. Materials and Methods

### 4.1. Study Design and Settings

A point prevalence survey of antimicrobial was undertaken across hospitals in Pakistan using the Global-PPS methodology from November 2020 to January 2021 [60,106]. The objective is to evaluate and document prevalence and prescribing patterns of antimicrobials among a number of hospitals during the current COVID-19 pandemic. This included patients with or without COVID-19 admitted to hospital. In total, 41 hospitals from secondary and tertiary care sectors, either private or public groups, were initially invited from the different cities throughout Pakistan to participate in this PPS using a purposeful sampling approach. We included both private and public hospitals to reflect the current situation in Pakistan, similar to PPS studies undertaken in other countries [107,108]. Participation of the hospitals was voluntary. Overall, twenty-one hospitals from different cities throughout Pakistan agreed to participate in this PPS study. However, bed occupancy of six secondary care hospitals was extremely low because of the current COVID-19 pandemic. Moreover, patients typically do not stay overnight in these settings. Consequently, these six hospitals were excluded from the final list of participating hospitals. Likewise, health care facilities having only nursing care, rehabilitation centers, or psychiatric centers were also excluded. Finally, 11 hospitals were included in this current PPS reflecting previous PPS studies in Pakistan [61,109].

### 4.2. Instrument of Measure

In order to collect data at the hospital, ward and patient level, uniform paper data collection forms were used from the Global PPS design [59,60]. The hospital information form in the Global PPS studies included the category of the participating hospital, the number of departments, the number of patients hospitalized and bed capacities. The medicine formulary of each hospital was checked before data collection with respect to antimicrobial availability in hospitals as there can be shortages, which is similar to other LMICs [110,111,112,113]. The ward data form consisted of total bed capacity, department specialty and the total number of patients that were admitted. The patient data form comprised their gender, prescribed antibiotics and their dosage regimen, the number of antimicrobials per patient, reasons for antibiotic prescribing and causative microorganisms if documented. A web-based program designed by University of Antwerp was used for data entry, validation and reporting [110].

With respect to guideline availability and adherence, antimicrobial guidelines have not been developed nationally within Pakistan or typically among the institutions surveyed. In their absence, institutions typically follow prescribing guidance for dosage and indications included in the British National Formulary [94,114]; alternatively, currently do not follow any guidelines. This is being addressed with national guidelines currently being developed through the National Institute of Health in Pakistan.

### 4.3. Inclusion and Exclusive Criteria

Patients who were receiving at least one antimicrobial (antibacterial, antifungal or antiviral) for their clinical condition for systemic use were included in the PPS. Short-stay patients, discharged patients and patients in emergency, outpatient departments and long-term care units were excluded in line with other PPS studies [69,109].

### 4.4. Data Collection

As mentioned, a structured data collection tool (Global PPS) was used to collect the data. Prescribing charts and patient’s medical case notes were checked for detailed information regarding the variables of interest. All patients hospitalized overnight and remained in the ward at 08:00 am on the day of the PPS were included. All the prescribed antimicrobials at the time of the survey were included. The data was double-checked for accuracy and completeness. All the definitions of medical treatment including surgical prophylaxis, healthcare associated infections (HAI) and community acquired infections were taken from Global PPS method [110]. There was no contact with any patient at any stage of data collection. The collected data were entered onto the web-based Global-PPS program. The Anatomical Therapeutic Chemical (ATC) classification system of the WHO was used to classify the different antimicrobials [115]. In addition, the principal antibiotics prescribed were broken down by their AWaRe classification, with the prescribing of antibiotics from the ‘Access’ list preferred to those from the ‘Watch’ and ‘Reserve’ list to reduce resistance potential [59,60,96,116]. 

### 4.5. Antimicrobial Stewardship Program Exemplars

A narrative review was undertaken by the co-authors to document exemplars of ASPs undertaken among LMICs and their outcome to provide future guidance. We have used this approach before when providing examples [16,94,105,117,118]. The LMICs will be broken down into their World Bank Classification, building on previous publications, since there have been concerns regarding the ability of LMICs to undertake ASPs due to resource issues [54,105,119]. However, this is now less of an issue [55,56].

### 4.6. Statistical Analysis

Data were analyzed using the Microsoft Excel and Statistical Package for the Social Sciences (SPSS) and descriptive statistics were applied.

## 5. Conclusions

There were concerns with high levels of prescribing of antibiotics among hospitals in Pakistan during the COVID-19 pandemic. Empiric prescribing dominated, with ceftriaxone the most commonly prescribed antibiotic. This is a concern as such prescribing may well enhance already high rates of AMR in Pakistan. Various strategies and initiatives are required to improve future prescribing of antibiotics among hospitals in Pakistan. This includes the routine introduction of ASPs among hospitals throughout Pakistan as well as increasing the capacity of hospitals to routinely undertake culture and sensitivity testing. ASPs can incorporate educational programs surrounding the rational prescribing of antibiotics, continuous supervision and feedback. Key areas for ASPs include increasing the documentation of the indication for prescribing, reducing empiric prescribing, reducing the extent of antibiotics prescribed post-operatively to prevent SSIs and increasing the prescribing of ‘Access’ antibiotics where pertinent as part of newly developed national guidelines. In addition, increasing the prescribing of oral antibiotics via de-escalation where applicable to reduce hospital stay and costs.

## Figures and Tables

**Table 1:** Overall antibiotic use prevalence.

Characteristics N (%)	Total
Total beds	1810
Hospitalized patients	1024
Number of treated patients	662 (64.64)
Number of prescribed antibiotics	1191 (1.76/patient)
**Departments**	
Surgical ward	286 (24)
Medical ward	500 (42)
Intensive care unit	67 (5.6)
Pediatric medical ward	331 (27.8)
Pediatric intensive care unit	7 (0.6)
**Gender**	
Male	681 (57.2)
Female	510 (42.8)
**Route of administration (where recorded)**	
Oral	89 (7.5)
Parenteral	1098 (92.2)
**Indication**	
Community-acquired infection	856 (71.9)
Hospital-acquired infection	20 (1.7)
Medical prophylaxis	30 (2.5)
Surgical prophylaxis (single dose)	5 (0.4)
Surgical prophylaxis (one day)	85 (7.1)
Surgical prophylaxis (>1 day)	163 (13.7)
Others	17 (1.4)
**Treatment**	
Empirical therapy	1166 (97.9)
Targeted therapy	25 (2.1)
**Guideline compliance**	
Yes	12 (1.0)
No	662 (55.6)
NA	466 (39.1)
NI	51 (4.3)
**Stop date documented**	118(9.9)
Reason on notes Yes No	122 (10.2) 1069 (89.8)
**Culture Reports**	1

Empirical therapy: Treatment given without finding out the causative microbe; Targeted therapy: Treatment given after finding the causative microbe; NA: Not assessable because of the absence of any guidelines; NI: No information because of incomplete patient history.

**Table 2:** Prevalence of the main antimicrobial classes.

Antibiotics	N (%)
**ANTIBACTERIALS FOR SYSTEMIC USE** (J01)	1163 (97.6)
Tetracyclines (J01A)	4 (0.3)
Amphenicols (J01B)	0 (0)
Penicillins (J01C)	147 (12.3)
Cephalosporins and carbapenems (J01D)	518 (43.5)
Sulfonamides and trimethoprim (J01E)	2 (0.2)
Macrolides and lincosamides (J01F)	54 (4.5)
Aminoglycosides (J01G)	129 (10.8)
Quinolones (J01M)	59 (5.0)
Other antibacterial (J01X)	247 (20.7)
ANTIMYCOTICS FOR SYSTEMIC USE (J02)	0 (0)
ANTIMYCOBACTERIALS FOR SYSTEMIC USE (J04)	11 (0.9)
ANTIVIRALS FOR SYSTEMIC USE (J05)	13 (1.1)
ANTIPROTOZOALS (P01)	0 (0)

**Table 3:** Top 10 indications and antibiotics.

	Top 10 Indications		Top 10 Antibiotics	
	Indications	N (%)	Antibiotics	N (%)
**1.**	Pneumonia	158 (13.3)	Ceftriaxone	317 (26.6)
**2.**	CNS	124(10.4)	Metronidazole	115 (9.7)
**3.**	GIT-P	124(10.4)	Vancomycin	94 (7.9)
**4.**	OBGY	121 (10.2)	Meropenem	66 (5.5)
**5.**	BJ	93 (7.8)	Ciprofloxacin	34 (2.9)
**6.**	CVS	78 (6.5)	Piperacillin, enzyme inhibitor	14(1.2)
**7.**	COVID-19	59 (5.0)	Levofloxacin	10(0.8)
**8.**	SST	50 (4.2)	Cefipime	6 (0.5)
**9.**	SEPSIS	27 (2.6)	Cefuroxime	4(0.3)
**10.**	Unknown	7 (0.6)	Clindamycin	2 (0.2)

BJ, bone and joint; CNS, central nervous system; GIT, gastro-intestinal tract; OBGY, obstetrics and gynecology; P, prophylaxis; SST, skin and soft tissues.

## Data Availability

Further data are available upon reasonable request from the corresponding authors.

## Supplementary Materials

The following supporting information can be downloaded at: https://www.mdpi.com/article/10.3390/antibiotics12010070/s1, Table S1: Examples of Antimicrobial Stewardship Programmes among LMICs and their impact. This includes [105,119,120,121,122,123,124,125,126,127,128,129,130,131,132,133,134].

## References

[1] Nicolaou K.C., Rigol S. (2018). A brief history of antibiotics and select advances in their synthesis. J. Antibiot..

[2] Zaffiri L., Gardner J., Toledo-Pereyra L.H. (2012). History of Antibiotics. From Salvarsan to Cephalosporins. J. Investig. Surg..

[3] McDermott W., Rogers D.E. (1982). Social ramifications of control of microbial disease. Johns Hopkins Med. J..

[4] Hamilton K.W. (2019). Miracle Cure: The Creation of Antibiotics and the Birth of Modern Medicine. Emerg. Infect. Dis..

[5] World Health Organization (2019). No Time to Wait: Securing the Future from Drug-Resistant Infections—Report to the Secretary-General of the United Nations. https://www.who.int/publications/i/item/no-time-to-wait-securing-the-future-from-drug-resistant-infections.

[6] Cassini A., Högberg L.D., Plachouras D., Quattrocchi A., Hoxha A., Simonsen G.S., Colomb-Cotinat M., Kretzschmar M.E., Devleesschauwer B., Cecchini M. (2019). Attributable deaths and disability-adjusted life-years caused by infections with antibiotic-resistant bacteria in the EU and the European Economic Area in 2015: A population-level modelling analysis. Lancet Infect. Dis..

[7] (2022). Global burden of bacterial antimicrobial resistance in 2019: A systematic analysis. Lancet.

[8] Majumder A.A., Rahman S., Cohall D., Bharatha A., Singh K., Haque M., Hilaire M.G.-S. (2020). Antimicrobial Stewardship: Fighting Antimicrobial Resistance and Protecting Global Public Health. Infect. Drug Resist..

[9] Llor C., Bjerrum L. (2014). Antimicrobial resistance: Risk associated with antibiotic overuse and initiatives to reduce the problem. Ther. Adv. Drug Saf..

[10] Bell B.G., Schellevis F., Stobberingh E., Goossens H., Pringle M. (2014). A systematic review and meta-analysis of the effects of antibiotic consumption on antibiotic resistance. BMC Infect. Dis..

[11] Munkholm L., Rubin O. (2020). The global governance of antimicrobial resistance: A cross-country study of alignment between the global action plan and national action plans. Glob. Health.

[12] World Bank Group (2019). Pulling Together to Beat Superbugs Knowledge and Implementation Gaps in Addressing Antimicrobial Resistance. https://openknowledge.worldbank.org/bitstream/handle/10986/32552/Pulling-Together-to-Beat-Superbugs-Knowledge-and-Implementation-Gaps-in-Addressing-Antimicrobial-Resistance.pdf?sequence=1&isAllowed=y.

[13] WHO (2015). Global Action Plan on Antimicrobial Resistance. https://apps.who.int/iris/bitstream/handle/10665/193736/9789241509763_eng.pdf?sequence=1.

[14] Sulis G., Adam P., Nafade V., Gore G., Daniels B., Daftary A., Das J., Gandra S., Pai M. (2020). Antibiotic prescription practices in primary care in low- and middle-income countries: A systematic review and meta-analysis. PLoS Med..

[15] Hadi M.A., Karami N.A., Al-Muwalid A.S., Al-Otabi A., Al-Subahi E., Bamomen A., Mohamed M.M.A., Elrggal M.E. (2016). Community pharmacists’ knowledge, attitude, and practices towards dispensing antibiotics without prescription (DAwP): A cross-sectional survey in Makkah Province, Saudi Arabia. Int. J. Infect. Dis..

[16] Godman B., Egwuenu A., Haque M., Malande O., Schellack N., Kumar S., Saleem Z., Sneddon J., Hoxha I., Islam S. (2021). Strategies to Improve Antimicrobial Utilization with a Special Focus on Developing Countries. Life.

[17] Zhu N., Zhang D., Wang W., Li X., Yang B., Song J., Zhao X., Huang B., Shi W., Lu R. (2020). A Novel Coronavirus from Patients with Pneumonia in China, 2019. N. Engl. J. Med..

[18] WHO (2022). Coronavirus COVID-19 Dashboard. https://covid19.who.int.

[19] Abid K., Bari Y.A., Younas M., Javaid S.T., Imran A. (2020). Progress of COVID-19 Epidemic in Pakistan. Asia Pac. J. Public Health.

[20] Mazhar S., Tanwir F. (2022). Pakistan’s Scenario in Pandemic Situation of COVID-19. J. Bahria Univ. Med. Dent. Coll..

[21] Akhtar H., Afridi M., Akhtar S., Ahmad H., Ali S., Khalid S., Awan S.M., Jahangiri S., Khader Y.S. (2021). Pakistan’s Response to COVID-19: Overcoming National and International Hypes to Fight the Pandemic. JMIR Public Health Surveill..

[22] Ul Mustafa Z., Salman M., Aldeyab M., Kow C.S., Hasan S.S. (2021). Antimicrobial consumption among hospitalized patients with COVID-19 in Pakistan. SN Compr. Clin. Med..

[23] Ayouni I., Maatoug J., Dhouib W., Zammit N., Ben Fredj S., Ghammam R., Ghannem H. (2021). Effective public health measures to mitigate the spread of COVID-19: A systematic review. BMC Public Health.

[24] Talic S., Shah S., Wild H., Gasevic D., Maharaj A., Ademi Z., Li X., Xu W., Mesa-Eguiagaray I., Rostron J. (2021). Effectiveness of public health measures in reducing the incidence of COVID-19, SARS-CoV-2 transmission, and COVID-19 mortality: Systematic review and meta-analysis. BMJ.

[25] Ameen L., Assaggaf H., Alsafi R., Minshawi F., Alghamdi S., Alharbi A., Qashqaric F., Makhdoomd H., Refaata B., Alsaife B. (2022). Analysis of the Clinical Characteristics of COVID-19 Patient Severity Amongst Saudi Hospital Admission in 2020. J. Umm Al-Qura Univ. Med. Sci..

[26] Al-Hadidi S.H., Alhussain H., Hadi H.A., Johar A., Yassine H.M., Al Thani A.A., Eltai N.O. (2021). The Spectrum of Antibiotic Prescribing During COVID-19 Pandemic: A Systematic Literature Review. Microb. Drug Resist..

[27] Langford B.J., So M., Raybardhan S., Leung V., Soucy J.-P.R., Westwood D., Daneman N., MacFadden D.R. (2021). Antibiotic prescribing in patients with COVID-19: Rapid review and meta-analysis. Clin. Microbiol. Infect..

[28] Langford B.J., So M., Raybardhan S., Leung V., Westwood D., MacFadden D.R., Soucy J.-P.R., Daneman N. (2020). Bacterial co-infection and secondary infection in patients with COVID-19: A living rapid review and meta-analysis. Clin. Microbiol. Infect..

[29] Baggs J., Rose A.N., McCarthy N.L., Wolford H., Srinivasan A., A Jernigan J., Reddy S.C. (2022). Antibiotic-Resistant Infections Among Inpatients with Coronavirus Disease 2019 (COVID-19) in US Hospitals. Clin. Infect. Dis..

[30] Baghdadi J.D., Coffey K.C., Adediran T., Goodman K.E., Pineles L., Magder L.S., O’Hara L.M., Pineles B.L., Nadimpalli G., Morgan D.J. (2021). Antibiotic Use and Bacterial Infection among Inpatients in the First Wave of COVID-19: A Retrospective Cohort Study of 64,691 Patients. Antimicrob. Agents Chemother..

[31] Goncalves Mendes Neto A., Lo K.B., Wattoo A., Salacup G., Pelayo J., DeJoy R., Bhargav R., Gul F., Peterson E., Albano J. (2021). Bacterial infections and patterns of antibiotic use in patients with COVID-19. J. Med. Virol..

[32] Mustafa Z.U., Tariq S., Iftikhar Z., Meyer J.C., Salman M., Mallhi T.H., Khan Y.H., Godman B., Seaton R.A. (2022). Predictors and Outcomes of Healthcare-Associated Infections among Patients with COVID-19 Admitted to Intensive Care Units in Punjab, Pakistan; Findings and Implications. Antibiotics.

[33] E Nelson R., Hatfield K.M., Wolford H., Samore M.H., Scott R.D., Reddy S.C., Olubajo B., Paul P., A Jernigan J., Baggs J. (2021). National Estimates of Healthcare Costs Associated with Multidrug-Resistant Bacterial Infections Among Hospitalized Patients in the United States. Clin. Infect. Dis..

[34] Guan W.-j., Ni Z.-y, Hu Y., Liang W.-h., Ou C.-q., He J.-x., Liu L., Shan H., Lei C.-l., Hui D.S.C. (2020). Clinical characteristics of coronavirus disease 2019 in China. N. Engl. J. Med..

[35] Teich V.D., Klajner S., de Almeida F.A.S., Dantas A.C.B., Laselva C.R., Torritesi M.G., Canero T.R., Berwanger O., Rizzo L.V., Reis E.P. (2020). Epidemiologic and clinical features of patients with COVID-19 in Brazil. Einstein.

[36] Rawson T.M., Moore L.S.P., Zhu N., Ranganathan N., Skolimowska K., Gilchrist M., Satta G., Cooke G., Holmes A.H. (2020). Bacterial and Fungal Coinfection in Individuals with Coronavirus: A Rapid Review to Support COVID-19 Antimicrobial Prescribing. Clin. Infect. Dis..

[37] Alshaikh F.S., Godman B., Sindi O.N., Seaton R.A., Kurdi A. (2022). Prevalence of bacterial coinfection and patterns of antibiotics prescribing in patients with COVID-19: A systematic review and meta-analysis. PLoS ONE.

[38] Tan S.H., Ng T.M., Tay H.L., Yap M.Y., Heng S.T., Loo A.Y.X., Teng C.B., Lee T.H. (2021). A point prevalence survey to assess antibiotic prescribing in patients hospitalized with confirmed and suspected coronavirus disease 2019 (COVID-19). J. Glob. Antimicrob. Resist..

[39] Lai C.-C., Chen S.-Y., Ko W.-C., Hsueh P.-R. (2021). Increased antimicrobial resistance during the COVID-19 pandemic. Int. J. Antimicrob. Agents.

[40] Rusic D., Vilovic M., Bukic J., Leskur D., Perisin A.S., Kumric M., Martinovic D., Petric A., Modun D., Bozic J. (2021). Implications of COVID-19 Pandemic on the Emergence of Antimicrobial Resistance: Adjusting the Response to Future Outbreaks. Life.

[41] Rossato L., Negrão F.J., Simionatto S. (2020). Could the COVID-19 pandemic aggravate antimicrobial resistance?. Am. J. Infect. Control.

[42] Hsu J. (2020). How COVID-19 is accelerating the threat of antimicrobial resistance. BMJ.

[43] Lucien M.A.B., Canarie M.F., Kilgore P.E., Jean-Denis G., Fénélon N., Pierre M., Cerpa M., Joseph G.A., Maki G., Zervos M.J. (2021). Antibiotics and antimicrobial resistance in the COVID-19 era: Perspective from resource-limited settings. Int. J. Infect. Dis..

[44] Jeon K., Jeong S., Lee N., Park M.-J., Song W., Kim H.-S., Kim H.S., Kim J.-S. (2022). Impact of COVID-19 on Antimicrobial Consumption and Spread of Multidrug-Resistance in Bacterial Infections. Antibiotics.

[45] Marua A.M., Shethwala N.D., Bhatt P., Shah A. (2022). Evaluation of Bacterial Co-Infections and Antibiotic Resistance in Positive COVID-19 Patients. Maedica.

[46] Getahun H., Smith I., Trivedi K., Paulin S., Balkhy H.H. (2020). Tackling antimicrobial resistance in the COVID-19 pandemic. Bull. World Health Organ..

[47] Molla M.A., Yeasmin M., Islam K., Sharif M., Amin R., Nafisa T., Ghosh A.K., Parveen M., Arif M.H., Alam J.A.J. (2021). Antibiotic Prescribing Patterns at COVID-19 Dedicated Wards in Bangladesh: Findings from a Single Center Study. Infect. Prev. Pract..

[48] Sheikh S., Vishwas G., Aggarwal M., Bhattacharya S., Kumari P., Parashar L., Meshram G. (2022). Antibiotic point prevalence survey at a tertiary healthcare hospital in India: Identifying strategies to improve the antibiotic stewardship program immediately after a COVID-19 wave. Infect. Prev. Pract..

[49] Apisarnthanarak A., Weber D.J. (2022). Strategy to limit multidrug-resistant *Acinetobacter baumannii* transmission in a cohort coronavirus disease 2019 (COVID-19) critical care unit. Infect. Control Hosp. Epidemiol..

[50] Ahmed N., Khan M., Saleem W., Karobari M.I., Mohamed R.N., Heboyan A., Rabaan A.A., Al Mutair A., Alhumaid S., Alsadiq S.A. (2022). Evaluation of Bi-Lateral Co-Infections and Antibiotic Resistance Rates among COVID-19 Patients. Antibiotics.

[51] Haseeb A., Faidah H.S., Al-Gethamy M., Iqbal M.S., Barnawi A.M., Elahe S.S., Bukhari D.N., Al-Sulaimani T.M.N., Fadaaq M., Alghamdi S. (2021). Evaluation of a Multidisciplinary Antimicrobial Stewardship Program in a Saudi Critical Care Unit: A Quasi-Experimental Study. Front. Pharmacol..

[52] Haseeb A., Faidah H.S., Alghamdi S., Alotaibi A.F., Elrggal M.E., Mahrous A.J., Abuhussain S.S.A., Obaid N.A., Algethamy M., AlQarni A. (2021). Dose Optimization of Colistin: A Systematic Review. Antibiotics.

[53] Nathwani D., Varghese D., Stephens J., Ansari W., Martin S., Charbonneau C. (2019). Value of hospital antimicrobial stewardship programs [ASPs]: A systematic review. Antimicrob. Resist. Infect. Control.

[54] Cox J.A., Vlieghe E., Mendelson M., Wertheim H., Ndegwa L., Villegas M.V., Gould I., Hara G.L. (2017). Antibiotic stewardship in low-and middle-income countries: The same but different?. Clin. Microbiol. Infect..

[55] Akpan M.R., Isemin N.U., Udoh A.E., Ashiru-Oredope D. (2020). Implementation of antimicrobial stewardship programmes in African countries: A systematic literature review. J. Glob. Antimicrob. Resist..

[56] Siachalinga L., Mufwambi W., Lee L.-H. (2022). Impact of antimicrobial stewardship interventions to improve antibiotic prescribing for hospital inpatients in Africa: A systematic review and meta-analysis. J. Hosp. Infect..

[57] Kalungia A.C., Mwambula H., Munkombwe D., Marshall S., Schellack N., May C., Jones A.S.C., Godman B. (2019). Antimicrobial stewardship knowledge and perception among physicians and pharmacists at leading tertiary teaching hospitals in Zambia: Implications for future policy and practice. J. Chemother..

[58] Fadare J.O., Ogunleye O., Iliyasu G., Adeoti A., Schellack N., Engler D., Massele A., Godman B. (2019). Status of antimicrobial stewardship programmes in Nigerian tertiary healthcare facilities: Findings and implications. J. Glob. Antimicrob. Resist..

[59] Pauwels I., Versporten A., Drapier N., Vlieghe E., Goossens H., Koraqi A., Hoxha I., Tafaj S., Cornistein W., Quiros R. (2021). Hospital antibiotic prescribing patterns in adult patients according to the WHO Access, Watch and Reserve classification (AWaRe): Results from a worldwide point prevalence survey in 69 countries. J. Antimicrob. Chemother..

[60] Versporten A., Zarb P., Caniaux I., Gros M.-F., Drapier N., Miller M., Jarlier V., Nathwani D., Goossens H., Koraqi A. (2018). Antimicrobial consumption and resistance in adult hospital inpatients in 53 countries: Results of an internet-based global point prevalence survey. Lancet Glob. Health.

[61] Saleem Z., Hassali M.A., Godman B., Versporten A., Hashmi F.K., Saeed H., Saleem F., Salman M., Rehman I.U., Khan T.M. (2020). Point prevalence surveys of antimicrobial use: A systematic review and the implications. Expert Rev. Anti-Infect. Ther..

[62] Haseeb A., Faidah H.S., Algethamy M., Alghamdi S., Alhazmi G.A., Alshomrani A.O., Alqethami B.R., Alotibi H.S., Almutiri M.Z., Almuqati K.S. (2021). Antimicrobial Usage and Resistance in Makkah Region Hospitals: A Regional Point Prevalence Survey of Public Hospitals. Int. J. Environ. Res. Public Health.

[63] WHO Therapeutics and COVID-19: Living Guideline—22 April 2022. COVID-19: Clinical Care. https://www.who.int/publications/i/item/WHO-2019-nCoV-therapeutics-2022.3.

[64] Sieswerda E., de Boer M.G., Bonten M.M., Boersma W.G., Jonkers R.E., Aleva R.M., Kullberg B.-J., Schouten J.A., van de Garde E.M., Verheij T.J. (2021). Recommendations for antibacterial therapy in adults with COVID-19—An evidence based guideline. Clin. Microbiol. Infect..

[65] Afriyie D.K., A Sefah I., Sneddon J., Malcolm W., McKinney R., Cooper L., Kurdi A., Godman B., Seaton R.A. (2020). Antimicrobial point prevalence surveys in two Ghanaian hospitals: Opportunities for antimicrobial stewardship. JAC-Antimicrob. Resist..

[66] Mustafa Z.U., Saleem M.S., Ikram M.N., Salman M., Butt S.A., Khan S., Godman B., Seaton R.A. (2022). Co-infections and antimicrobial use among hospitalized COVID-19 patients in Punjab, Pakistan: Findings from a multicenter, point prevalence survey. Pathog. Glob. Health.

[67] Ramzan K., Shafiq S., Raees I., Mustafa Z.U., Salman M., Khan A.H., Meyer J.C., Godman B. (2022). Co-Infections, Secondary Infections, and Antimicrobial Use in Patients Hospitalized with COVID-19 during the First Five Waves of the Pandemic in Pakistan; Findings and Implications. Antibiotics.

[68] Saleem Z., Hassali M.A., Hashmi F.K., Godman B., Bhutta O.A. (2019). A repeated point prevalence survey of antimicrobial use in specialized cancer care hospital of Pakistan: Findings and implications. Hosp. Pract..

[69] Saleem Z., Hassali M.A., Versporten A., Godman B., Hashmi F.K., Goossens H., Saleem F. (2019). A multicenter point prevalence survey of antibiotic use in Punjab, Pakistan: Findings and implications. Expert Rev. Anti-Infect. Ther..

[70] Saleem Z., Godman B., Azhar F., Kalungia A.C., Fadare J., Opanga S., Markovic-Pekovic V., Hoxha I., Saeed A., Al-Gethamy M. (2022). Progress on the national action plan of Pakistan on antimicrobial resistance (AMR): A narrative review and the implications. Expert Rev. Anti-Infect. Ther..

[71] Saleem Z., Saeed H., Hassali M.A., Godman B., Asif U., Yousaf M., Ahmed Z., Riaz H., Raza S.A. (2019). Pattern of inappropriate antibiotic use among hospitalized patients in Pakistan: A longitudinal surveillance and implications. Expert. Rev. Anti-Infect. Ther..

[72] Saleem Z., Hassali M.A., Godman B., Hashmi F.K., Saleem F. (2019). Antimicrobial prescribing and determinants of antimicrobial resistance: A qualitative study among physicians in Pakistan. Int. J. Clin. Pharm..

[73] Saleem Z., Hassali M.A., Godman B., Fatima M., Ahmad Z., Sajid A., Rehman I.U., Nadeem M.U., Javaid Z., Malik M. (2020). Sale of WHO AWaRe groups antibiotics without a prescription in Pakistan: A simulated client study. J. Pharm. Policy Pract..

[74] Saleem Z., Hassali M.A., Hashmi F.K., Godman B., Ahmed Z. (2019). Snapshot of antimicrobial stewardship programs in the hospitals of Pakistan: Findings and implications. Heliyon.

[75] Ahmad T., Khan F.U., Ali S., Rahman A.U., Khan S.A. (2022). Assessment of without prescription antibiotic dispensing at community pharmacies in Hazara Division, Pakistan: A simulated client’s study. PLoS ONE.

[76] Mah E.M.S., Hassan M.Z., Biswas M., Rahman F., Akhtar Z., Das P., Islam M.A., Chowdhury F. (2021). Use of Antimicrobials among Suspected COVID-19 Patients at Selected Hospitals, Bangladesh: Findings from the First Wave of COVID-19 Pandemic. Antibiotics.

[77] Seaton R.A., Gibbons C.L., Cooper L., Malcolm W., McKinney R., Dundas S., Griffith D., Jeffreys D., Hamilton K., Choo-Kang B. (2020). Survey of antibiotic and antifungal prescribing in patients with suspected and confirmed COVID-19 in Scottish hospitals. J. Infect..

[78] Atif M., Azeem M., Saqib A., Scahill S. (2017). Investigation of antimicrobial use at a tertiary care hospital in Southern Punjab, Pakistan using WHO methodology. Antimicrob. Resist. Infect. Control.

[79] Oztoprak N., Cevik M.A., Akinci E., Korkmaz M., Erbay A., Eren S.S., Balaban N., Bodur H. (2006). Risk factors for ICU-acquired methicillin-resistant Staphylococcus aureus infections. Am. J. Infect. Control.

[80] Wolkewitz M., Vonberg R.P., Grundmann H., Beyersmann J., Gastmeier P., Bärwolff S., Geffers C., Behnke M., Rüden H., Schumacher M. (2008). Risk factors for the development of nosocomial pneumonia and mortality on intensive care units: Application of competing risks models. Crit. Care.

[81] Rees E.M., Nightingale E.S., Jafari Y., Waterlow N.R., Clifford S., Pearson C.A.B., Jombart T., Procter S.R., Knight G.M., CMMID Working Group (2020). COVID-19 length of hospital stay: A systematic review and data synthesis. BMC Med..

[82] Lansbury L., Lim B., Baskaran V., Lim W.S. (2020). Co-infections in people with COVID-19: A systematic review and meta-analysis. J. Infect..

[83] Amponsah O.K.O., Buabeng K.O., Owusu-Ofori A., Ayisi-Boateng N.K., Hämeen-Anttila K., Enlund H. (2021). Point prevalence survey of antibiotic consumption across three hospitals in Ghana. JAC-Antimicrob. Resist..

[84] Kumar S., Haque M., Shetty A., Choudhary S., Bhatt R., Sinha V., Manohar B., Chowdhury K., Nusrat N., Jahan N. (2022). Characteristics and Management of Children with Suspected COVID-19 Admitted to Hospitals in India: Implications for Future Care. Cureus.

[85] Ogunleye O.O., Oyawole M.R., Odunuga P.T., Kalejaye F., Yinka-Ogunleye A.F., Olalekan A., Ogundele S.O., Ebruke B.E., Richard A.K., Paramadhas B.D.A. (2022). A multicentre point prevalence study of antibiotics utilization in hospitalized patients in an urban secondary and a tertiary healthcare facilities in Nigeria: Findings and implications. Expert Rev. Anti-Infect. Ther..

[86] Kurdi A., Hasan A.J., Baker K.I., Seaton R.A., Ramzi Z.S., Sneddon J., Godman B. (2021). A multicentre point prevalence survey of hospital antibiotic prescribing and quality indices in the Kurdistan regional government of Northern Iraq: The need for urgent action. Expert Rev. Anti-Infect. Ther..

[87] Vercheval C., Gillet M., Maes N., Albert A., Frippiat F., Damas P., Van Hees T. (2016). Quality of documentation on antibiotic therapy in medical records: Evaluation of combined interventions in a teaching hospital by repeated point prevalence survey. Eur. J. Clin. Microbiol..

[88] Yeo J.M. (2016). Antimicrobial stewardship: Improving antibiotic prescribing practice in a respiratory ward. BMJ Open Qual..

[89] Junaid E., Jenkins L., Swanepoel H., North Z., Gould T. (2018). Antimicrobial stewardship in a rural regional hospital—Growing a positive culture. S. Afr. Med. J..

[90] Gitaka J., Kamita M., Mureithi D., Ndegwa D., Masika M., Omuse G., Ngari M., Makokha F., Mwaura P., Mathai R. (2020). Combating antibiotic resistance using guidelines and enhanced stewardship in Kenya: A protocol for an implementation science approach. BMJ Open.

[91] Foxlee N.D., Townell N., Heney C., McIver L., Lau C.L. (2021). Strategies Used for Implementing and Promoting Adherence to Antibiotic Guidelines in Low- and Lower-Middle-Income Countries: A Systematic Review. Trop. Med. Infect. Dis..

[92] Nampoothiri V., Sudhir A., Joseph M., Mohamed Z., Menon V., Charani E., Singh S. (2021). Mapping the Implementation of a Clinical Pharmacist-Driven Antimicrobial Stewardship Programme at a Tertiary Care Centre in South India. Antibiotics.

[93] Campbell S.M., Meyer J., Godman B. (2021). Why Compliance to National Prescribing Guidelines is Important Especially across Sub-Saharan Africa and Suggestions for the Future. Biomed. Pharm. Sci..

[94] Mwita J.C., Ogunleye O.O., Olalekan A., Kalungia A.C., Kurdi A., Saleem Z., Sneddon J., Godman B. (2021). Key Issues Surrounding Appropriate Antibiotic Use for Prevention of Surgical Site Infections in Low- and Middle-Income Countries: A Narrative Review and the Implications. Int. J. Gen. Med..

[95] Sharland M., Gandra S., Huttner B., Moja L., Pulcini C., Zeng M., Mendelson M., Cappello B., Cooke G., Magrini N. (2019). Encouraging AWaRe-ness and discouraging inappropriate antibiotic use—The new 2019 Essential Medicines List becomes a global antibiotic stewardship tool. Lancet Infect. Dis..

[96] Hsia Y., Sharland M., Jackson C., Wong I.C.K., Magrini N., A Bielicki J. (2019). Consumption of oral antibiotic formulations for young children according to the WHO Access, Watch, Reserve (AWaRe) antibiotic groups: An analysis of sales data from 70 middle-income and high-income countries. Lancet Infect. Dis..

[97] Yousif M.M.A. (2015). The Prevalence of Extended Spectrum β-Lactamase and Amp C-Producing Bacteria in a Sudanese Tertiary Hospital. Sudan Med. J..

[98] Khan F.A., Singh V.K., Sharma S., Singh P. (2013). A prospective study on the antimicrobial usage in the medicine department of a tertiary care teaching hospital. J. Clin. Diagn. Res. JCDR.

[99] Kiguba R., Karamagi C., Bird S.M. (2016). Extensive antibiotic prescription rate among hospitalized patients in Uganda: But with frequent missed-dose days. J. Antimicrob. Chemother..

[100] Nestler M.J., Godbout E., Lee K., Kim J., Noda A.J., Taylor P., Pryor R., Markley J.D., Doll M., Bearman G. (2021). Impact of COVID-19 on pneumonia-focused antibiotic use at an academic medical center. Infect. Control Hosp. Epidemiol..

[101] Aldeyab M.A., Harbarth S., Vernaz N., Kearney M.P., Scott M.G., Elhajji F.W.D., Aldiab M.A., McElnay J.C. (2012). The impact of antibiotic use on the incidence and resistance pattern of extended-spectrum beta-lactamase-producing bacteria in primary and secondary healthcare settings. Br. J. Clin. Pharmacol..

[102] Almeleebia T.M., Alhifany A.A., Almutairi F., Alshibani M., Alhossan A.M. (2021). Regulating antimicrobial sales in Saudi Arabia: Achievements and challenges. Int. J. Clin. Pract..

[103] Almangour T.A., Alenazi B., Ghonem L., Alhifany A.A., Aldakheel B.A., Alruwaili A. (2020). Inhaled colistin for the treatment of nosocomial pneumonia due to multidrug-resistant Gram-negative bacteria: A real-life experience in tertiary care hospitals in Saudi Arabia. Saudi Pharm J..

[104] Almangour T.A., Ghonem L., Aljabri A., Alruwaili A., Al Musawa M., Damfu N., Almalki M.S., Alattas M., Abed H., Naeem D. (2022). Ceftazidime-Avibactam versus Colistin for the Treatment of Infections Due to Carbapenem-Resistant Enterobacterales: A Multicenter Cohort Study. Infect. Drug Resist..

[105] Saleem Z., Godman B., Cook A., Khan M.A., Campbell S.M., Seaton R.A., Siachalinga L., Haseeb A., Amir A., Kurdi A. (2022). Ongoing Efforts to Improve Antimicrobial Utilization in Hospitals among African Countries and Implications for the Future. Antibiotics.

[106] Rachina S., Belkova Y., Kozlov R., Versporten A., Pauwels I., Goossens H., Bochanova E., Domanskaya O., Elokhina E., Ezhova L. (2020). Longitudinal Point Prevalence Survey of Antimicrobial Consumption in Russian Hospitals: Results of the Global-PPS Project. Antibiotics.

[107] Anand Paramadhas B.D., Tiroyakgosi C., Mpinda-Joseph P., Morokotso M., Matome M., Sinkala F., Gaolebe M., Malone B., Molosiwa E., Shanmugam M.G. (2019). Point prevalence study of antimicrobial use among hospitals across Botswana; findings and implications. Expert. Rev. Anti Infect. Ther..

[108] Chowdhury K., Haque M., Nusrat N., Adnan N., Islam S., Lutfor A.B., Begum D., Rabbany A., Karim E., Malek A. (2022). Management of Children Admitted to Hospitals across Bangladesh with Suspected or Confirmed COVID-19 and the Implications for the Future: A Nationwide Cross-Sectional Study. Antibiotics.

[109] Arif S., Sadeeqa S., Saleem Z. (2021). Patterns of Antimicrobial Use in Hospitalized Children: A Repeated Point Prevalence Survey from Pakistan. J. Pediatr. Infect. Dis. Soc..

[110] Global PPS Global Point Prevalence Survey. http://www.global-pps.com/.

[111] Atif M., Malik I., Mushtaq I., Asghar S. (2019). Medicines shortages in Pakistan: A qualitative study to explore current situation, reasons and possible solutions to overcome the barriers. BMJ Open.

[112] Acosta A., Vanegas E.P., Rovira J., Godman B., Bochenek T. (2019). Medicine Shortages: Gaps Between Countries and Global Perspectives. Front. Pharmacol..

[113] Chigome A.K., Matlala M., Godman B., Meyer J.C. (2019). Availability and use of therapeutic interchange policies in managing antimicrobial shortages among South African public sector hospitals; findings and implications. Antibiotics.

[114] García-Vello P., Brobbey F., González-Zorn B., Saba C.K.S. (2020). A cross-sectional study on antibiotic prescription in a teaching hospital in Ghana. Pan Afr. Med. J..

[115] WHO (2021). Anatomical Therapeutic Chemical (ATC) Classification. https://www.who.int/tools/atc-ddd-toolkit/atc-classification.

[116] Hsia Y., Lee B.R., Versporten A., Yang Y., Bielicki J., Jackson C., Newland J., Goossens H., Magrini N., Sharland M. (2019). Use of the WHO Access, Watch, and Reserve classification to define patterns of hospital antibiotic use (AWaRe): An analysis of paediatric survey data from 56 countries. Lancet Glob. Health.

[117] Godman B., Haque M., McKimm J., Abu Bakar M., Sneddon J., Wale J., Campbell S., Martin A.P., Hoxha I., Abilova V. (2020). Ongoing strategies to improve the management of upper respiratory tract infections and reduce inappropriate antibiotic use particularly among lower and middle-income countries: Findings and implications for the future. Curr. Med. Res. Opin..

[118] Godman B., Egwuenu A., Wesangula E., Schellack N., Kalungia A.C., Tiroyakgosi C., Kgatlwane J., Mwita J.C., Patrick O., Niba L.L. (2022). Tackling antimicrobial resistance across sub-Saharan Africa: Current challenges and implications for the future. Expert Opin. Drug Saf..

[119] Gebretekle G.B., Mariam D.H., Taye W.A., Fentie A.M., Degu W.A., Alemayehu T., Beyene T., Libman M., Fenta T.G., Yansouni C.P. (2020). Half of Prescribed Antibiotics Are Not Needed: A Pharmacist-Led Antimicrobial Stewardship Intervention and Clinical Outcomes in a Referral Hospital in Ethiopia. Front. Public Health.

[120] Alabi A.S., Picka S.W., Sirleaf R., Ntirenganya P.R., Ayebare A., Correa N., Anyango S., Ekwen G., Agu E., Cook R. (2022). Implementation of an antimicrobial stewardship programme in three regional hospitals in the south-east of Liberia: Lessons learned. JAC-Antimicrob. Resist..

[121] Lester R., Haigh K., Wood A., E MacPherson E., Maheswaran H., Bogue P., Hanger S., Kalizang’Oma A., Srirathan V., Kulapani D. (2020). Sustained Reduction in Third-generation Cephalosporin Usage in Adult Inpatients Following Introduction of an Antimicrobial Stewardship Program in a Large, Urban Hospital in Malawi. Clin. Infect. Dis..

[122] Gentilotti E., De Nardo P., Nguhuni B., Piscini A., Damian C., Vairo F., Chaula Z., Mencarini P., Torokaa P., Zumla A. (2020). Implementing a combined infection prevention and control with antimicrobial stewardship joint program to prevent caesarean section surgical site infections and antimicrobial resistance: A Tanzanian tertiary hospital experience. Antimicrob. Resist. Infect. Control.

[123] Shankar R. (2018). Implementation of the WHO Surgical Safety Checklist at a teaching hospital in India and evaluation of the effects on perioperative complications. Int. J. Health Plan. Manag..

[124] Ayieko P., Irimu G., Ogero M., Mwaniki P., Malla L., Julius T., Chepkirui M., Mbevi G., Oliwa J., Agweyu A. (2019). Effect of enhancing audit and feedback on uptake of childhood pneumonia treatment policy in hospitals that are part of a clinical network: A cluster randomized trial. Implement. Sci..

[125] Allegranzi B., Aiken A.M., Kubilay N.Z., Nthumba P., Barasa J., Okumu G., Mugarura R., Elobu A.E., Jombwe J., Maimbo M. (2018). A multimodal infection control and patient safety intervention to reduce surgical site infections in Africa: A multicentre, before–after, cohort study. Lancet Infect. Dis..

[126] Kim R.Y., Kwakye G., Kwok A.C., Baltaga R., Ciobanu G., Merry A.F., Funk L., Lipsitz S., Gawande A., Berry W. (2015). Sustainability and long-term effectiveness of the WHO surgical safety checklist combined with pulse oximetry in a resource-limited setting: Two-year update from Moldova. JAMA Surg..

[127] Butt S.Z., Ahmad M., Saeed H., Saleem Z., Javaid Z. (2019). Post-surgical antibiotic prophylaxis: Impact of pharmacist’s educational intervention on appropriate use of antibiotics. J. Infect. Public Health.

[128] Yang Z., Zhao P., Wang J., Tong L., Cao J., Tian Y., Yao Z., Wang J., Zhu Y., Jia Y. (2014). DRUGS system enhancing adherence of Chinese surgeons to antibiotic use guidelines during perioperative period. PLoS ONE.

[129] Mahmoudi L., Ghouchani M., Mahi-Birjand M., Bananzadeh A., Akbari A. (2019). Optimizing compliance with surgical antimicrobial prophylaxis guidelines in patients undergoing gastrointestinal surgery at a referral teaching hospital in southern Iran: Clinical and economic impact. Infect Drug Resist..

[130] Mardani M., Abolghasemi S., Shabani S. (2020). Impact of an antimicrobial stewardship program in the antimicrobial-resistant and prevalence of clostridioides difficile infection and amount of antimicrobial consumed in cancer patients. BMC Res. Notes.

[131] Boyles T.H., Naicker V., Rawoot N., Raubenheimer P.J., Eick B., Mendelson M. (2017). Sustained reduction in antibiotic consumption in a South African public sector hospital; Four year outcomes from the Groote Schuur Hospital antibiotic stewardship program. S. Afr. Med. J..

[132] Bashar M.A., Miot J., Shoul E., van Zyl R.L. (2021). Impact of an antibiotic stewardship programme in a surgical setting. South. Afr. J. Infect. Dis..

[133] Apisarnthanarak A., Lapcharoen P., Vanichkul P., Srisaeng-Ngoen T., Mundy L.M. (2015). Design and analysis of a pharmacist-enhanced antimicrobial stewardship program in Thailand. Am. J. Infect. Control.

[134] Bozkurt F., Kaya S., Tekin R., Gulsun S., Deveci O., Dayan S., Hoşoglu S. (2014). Analysis of antimicrobial consumption and cost in a teaching hospital. J. Infect. Public Health.

