# Supplementary material for: Point Prevalence Survey of Antimicrobial Use during the COVID-19 Pandemic among Different Hospitals in Pakistan: Findings and Implications
Source: Antibiotics (Basel). 2022 Dec 30;12(1):70. doi: 10.3390/antibiotics12010070 (PMC9854885; doi:10.3390/antibiotics12010070)
Supplement: Supplementary file 1 [file antibiotics-12-00070-s001.zip › antibiotics-2075293-supplementary.pdf]

Supplementary Material: **Table S1** – Examples of Antimicrobial Stewardship Programmes among LMICs and their impact

Author, country and year	Brief details of the intervention	Impact of the Intervention
**Low-Income Countries***		
Ethiopia - Gebretekle et al, 2020 [119]	Principally Education. This included:<br>• Intervention - weekly audit meetings and immediate (verbal and written) feedback sessions regarding antibiotic prescriptions of admitted patients on 4 wards - building on recently developed institutional guidelines and training sessions with relevant clinicians on ASPs and guidelines<br>• Post-intervention phase – auditing of antibiotic prescriptions; however, no feedback to remind physicians	• Ceftriaxone, cefepime, meropenem, metronidazole and vancomycin were the most commonly prescribed antibiotics<br>• 96% of the recommendations made by the AMS team were accepted<br>• Once the intervention ceased, total antimicrobial use increased by 51.6% and the mean duration of treatment by 4.1 days/patient respectively<br>• Mean hospital stay as well as crude mortality also increased significantly after the intervention
Liberia - Alabi et al, 2022 [120]	• Education and Engineering involving a collation of three activities including the production and dissemination of local treatment guidelines, training and regular AMS ward rounds as well as monitoring agreed QIs<br>• QIs included prescribing of correct antibiotics (incorporating completeness of microbiological diagnostics) as well as dosages and duration<br>• QIs were assessed in a case series after AMS ward rounds and fed back to key personnel to further improve antibiotic prescribing	Improvements were seen in all QIs:<br>• Adherence to local guidelines improved from 34.5% to 61.0% (P<0.0005)<br>• Correct dosing improved from 15.2% to 36.5% (P<0.0005)<br>• Optimal duration of antibiotic use improved from 13.2% to 31.0% (P<0.0005)<br>• Proportion of patients receiving ceftriaxone reduced from 51.3% to 14.2% (P<0.0005).<br>• Following the ASP, 79.7% of patients now have samples sent for microbiological analysis
Malawi, Lester et al, 2020 [121]	• Education and Engineering involving guidelines, posters and the application of smartphones to help with clinical decision making as well as regular PPS studies combined with prescriber feedback<br>• The objective was to reduce extensive prescribing of third-generation cephalosporins within the hospital and associated costs with no adverse impact on mortality – especially with high rates of HIV among in-patients in the hospital (approximately 61% across the surveys)	• The proportion of prescriptions for an IV 3rd generation cephalosporin fell from 80.1% of all prescriptions at the first survey to 53.6% by the last survey<br>• The median length of a ceftriaxone course was reduced from 5 to 4 days aided by an increase in the number of clinician reviews of prescriptions at 48-hours increasing from 22.4% at the start to 73.3% by the final antibiotic survey<br>• Annual savings were estimated at US$15,000 with no change in mortality or median length of hospital stay
Tanzania, Gentilotti et al, 2020 [122]	• Multiple activities including formal and on-the-job training including seminars on infection prevention and control/ evidence-based education on AMR and good antimicrobial prescribing practice	• Pre-incision antibiotic prophylaxis for SAP was administered in significantly more cases post intervention (p < 0.001)

	• The objective of the educational activities was to enhance appropriate antibiotic prescribing to prevent SSIs for patients undergoing caesarean sections<br>• Prior to this - antibiotics were typically prescribed post-operatively (98.2%) and for 8–10 days when prescribed	• The extent of post-operative antibiotics for SAP was also appreciably lower post intervention ($p < 0.001$)<br>• The total number of SSIs decreased with appropriate use of antibiotics from 48% pre-intervention to 17% post intervention ($p < 0.001$)
**Low-Middle Income Countries***		
India, Shankar, 2018 [123]	Activities included:<br>• An agreement among key stakeholders regarding the key elements of the WHO checklist to help reduce SSIs<br>• A designated checklist coordinator worked with the surgical teams to confirm that the team had completed one task before proceeding to the next steps	• Prior to the implementation of this co-ordinated activities, all patients operated on a particular day were administered antibiotics in the morning irrespective of the timing of their surgery<br>• The correct practice of administering antibiotics within 1 to 2 hours of the incision was seen among all surgical patients following the intervention<br>• In addition, the rectification of any concerns that appropriate surgical patients were not being given SAP
Kenya, Ayieko et al, 2019 [124]	• Multiple activities with two groups<br>• Both groups received a half-day training on the new Kenyan pneumonia guidelines, with physicians in all hospitals supplied with updated protocol booklets including specific pneumonia algorithms. All hospitals also received continued network support<br>• The two groups were (i) Standard feedback with regular auditing and feedback of general paediatric care bimonthly and (ii) Enhanced feedback group – Regular auditing of agreed indicators of pneumonia care, with feedback delivered every month using specific feedback sheets<br>• The primary outcome measure was the proportion of all admitted patients with pneumonia who were correctly classified and treated within the first 24 hour period	• An improvement was seen in the enhanced feedback group concerning the correct classification and treatment of pneumonia after each round of enhanced feedback<br>• However, the performance declined in the standard feedback arm over time<br>• This was attributable to consistently poor performances among four out of the six participating facilities
Kenya, Uganda, Zambia, and Zimbabwe, Allegranzi et al, 2018 [125]	Multiple activities were undertaken to improve antibiotic prescribing for SAP. Activities included:<br>• Five planned visits to each participating hospital among the four participating African countries during the study period - supported by a range of educational tools<br>• Local teams identified key areas of concern with preventing SSIs; subsequently monitoring an agreed set of	• The appropriate use of antibiotics for SAP improved from 12.8% (baseline) to 39.1% of patients ($p < 0{\cdot}0001$) among the studied hospitals<br>• Concurrently, the cumulative incidence of SSIs decreased from a baseline of 8.0% to 3.8% post intervention ($p < 0{\cdot}0001$)

	quality indicators (six pre-identified ones including skin preparation and optimal timing of prophylaxis)<br>• Subsequent launch of pertinent tools and agreed quality indicators alongside monitoring/ feedback to improve future prescribing	
Moldova, Kim et al, 2015 [126]	Initiatives involved:<br>The introduction of a surgical safety checklist in the operating theatre over a week-long period<br>A data collection team developed the checklist and were randomly assigned to observe 30% of the surgical cases<br>Subsequently monitor agreed process adherence measures and feedback the findings to the surgical team	• 12.7% increase in the appropriate use of prophylactic antibiotics
Pakistan, Butt et al, 2019 [127]	Activities included:<br>• The results from pre-intervention studies regarding the prescribing of antibiotics for SAP were shared with physicians and nurses<br>• Subsequently general and specific problems regarding appropriate SAP were discussed with healthcare professionals and committees to enhance future adherence rates<br>• Training programmes over 10 to 15 days were conducted by hospital pharmacists for physicians and nurses to improve future antimicrobial use for SAP	• The appropriateness of prophylactic antibiotics increased from 11.6% to 28%<br>• Only 33% of patients received extended prophylaxis, i.e., > 60 hours, down from 42.9% of patients during the pre-intervention period (p=0.032)<br>• As a result, the costs of antibiotics significantly reduced (p=0.023)<br>• The average length of hospital stay fell from 4.50 days post intervention vs. 5.4 days pre-intervention - further reducing costs
**Upper-Middle Income Countries***		
China, Yang et al, 2014 [128]	• Multiple interventions to improve antibiotic use for SSIs<br>• These included the Introduction of a Drug Rational Usage Guideline System (DRUGS) vs. paper-based guidelines to enhance adherence to SAP guidelines	• Timing of the initial dose of antibiotic administration improved from 32.9% of patients - with antibiotics instigated within 30 min to 2 hours pre-incision - to 85.8% post intervention (statistically significant)<br>• The average length of hospital stay decreased from 7.00 days with paper-based guidelines to 2.55 days with DRUGS<br>• The average cost of antibiotics prescribed decreased from ¥3481 with paper-based guidelines to ¥1693 with DRUGS
Iran, Mahmoudi et al, 2019 [129]	Activities included:<br>• Revising SAP guidelines following meetings the surgical department and pharmacists, with senior clinical pharmacists delivering lectures about SAP to key members of the surgical teams<br>• Clinical pharmacists participating in ward rounds, attending recovery rooms	• The rate of antibiotic prescribing beyond 48 hours appreciably reduced to just 5.7% of patients - down from 92.1% of patients pre-intervention<br>• The appropriateness of antibiotic use increased to 91.4% of patients - up from 30.1% pre-intervention

	and communicated with surgeons when agreed guidelines were not being followed<br>• In addition, clinical pharmacists provided educational material to the surgical teams regarding SAP to enhance future care<br>• The rationality of SAP was continually evaluated during the perioperative period in accordance with agreed guidelines - with clinical pharmacists communicated any concerns with surgeons to improve future SAP	• The mean cost of antibiotics decreased more than 11-fold post intervention<br>• Th length of hospital stay reduced from an average of 5.14 days pre-intervention to 4.33 days (p<0.001) post intervention
Iran, Mardani et al, 2020 [130]	Activities included:<br>• Continuous educational programs for nurses and physicians employed in different wards of the hospital to improve future antimicrobial prescribing<br>• An inter-disciplinary ASP team performed a weekly scrutiny of the treatment of patients in the hospital based on their electronic medical records<br>• They subsequently provided feedback to appropriate nurses and physicians including their impact with reducing multiple antibiotic use and dosage changes	• A reduction in MDR cases in the year post intervention from 145 and 75 (p = 0.011)<br>• A significant reduction in all positive blood cultures (p = 0.001)<br>• A significant reduction in meropenem use (p = 0.043) as well as a significant reduction generally in antibiotic consumption, MDR organisms and CDIs
South Africa, Boyles et al, 2017 [131]	Key activities included:<br>• A comprehensive ASP programme comprising online education, a dedicated antibiotic prescription chart and weekly dedicated ward rounds to discuss current prescribing practices among key healthcare professionals – continued over 4 years<br>• Pre- and post-intervention data compared to provide future guidance	• Total antibiotic consumption fell from 1,046 DDDs/1 000 patient days (pre-intervention) to 868 (first 2 years of the interventoon - remaining at similar levels for the next 2 years)<br>• The improvements were driven by reductions in IV antibiotic use, particularly ceftriaxone<br>• Laboratory testing also increased over the same period<br>• Cost savings on antibiotics (inflation adjusted) were ZAR3.2 million over 4 years<br>• There was no significant change in mortality or 30-day readmission rates over the 4 years despi
South Africa, Bashar et al, 2021 [132]	Activities included:<br>• Regular ASP ward rounds on two surgical wards within the hospital<br>• During the ward rounds - each patient condition was discussed especially concerning antibiotic selection and laboratory investigations<br>• Potential switching from intravenous to oral agents, dose optimisation and any dose adjustments espeically in patients	• A reduction in the volume of antibiotic consumption from 739.30 DDDs/1000 to 564.93 DDDs/1000 patient days following the ASP<br>• Reduction in inappropriate antibiotic use from 35% to 26%<br>• An overall increase in culture targeted therapy<br>• Reduction in antibiotic administration for more than one day post operatively to prevent SSIs (from 7.3% to 6.6%)

	with renal and hepatic impairment were also discussed	Small (non-significant reduction) in total antibiotics administered IV (from 89.4% to 84.2%) alongside an increase in appropriate IV administration from 56.9% to 60.8%
Thailand, Apisarnthanarak et al, 2015 [133]	• The programme principally involved education with a 12-hour training course run by infectious diseases clinical pharmacists (IDCPs) with physicians looking after patients in medical wards<br>• This was coupled with an option for infectious diseases consultations (IDCs), daily rounds with ICDPs, or both if wished<br>• There was a control (Usual Standard of Care) group	Among patients with input from the IDCP group or the IDCP plus IDC group vs. controls, outcomes of the interventions were:<br>• Patients were less likely to be prescribed antibiotics inappropriate (P < .001)<br>• They had greater de-escalation of antibiotics (P < .001)<br>• More patients received antibiotics <7 days (P < .001)<br>• They had shorter lengths of stay (P < .001)
Turkey, Bozkurt et al, 2014 [134]	• Principally education involving a series of meetings with physicians from each clinic organised by the Infection Control Committee in the hospital to improve the prescribing of antimicrobials for SAP<br>• Alongside this, daily visits from the Infection Control Nurse as well as regular visits (twice per week) from an Infectious Diseases Control Specialist – more if compliance with agreed guidelines was seen a low<br>• Observations regularly shared with the physicians to improve future prescribing	• Use of appropriate antibiotics for SAP increased from 51% to 63.4% of cases<br>• Duration of use, i.e., limiting extended prophylaxis, improved from 10.3% to 59.4% of cases<br>• Total cost of antibiotics in the medical units, surgical units and ICUs decreased by 32.5%, 38.6% and 11.1% respectively

AMR: Antimicrobial Resistance; ASP: Antimicrobial Stewardship Programmes; DDD: Defined Daily Doses; MDR = Multidrug Resistant; SAP: Surgical Antibiotic Prophylaxis; *World Bank Classification [105,119]
